# Dual schwannomas of the ulnar nerve: the importance of surgical exploration beyond imaging: a case report

**DOI:** 10.1093/jscr/rjaf767

**Published:** 2025-12-28

**Authors:** Jose Antonio Prieto Meré, Valeria Mayorga Mata

**Affiliations:** Orthopedic Clinic, Edificio Integra Medical Center, 9A calle 4-52, Guatemala City 01010, Guatemala; Facultad de Medicina, Universidad Francisco Marroquin, 6ta avenida, 7-55 zona 10, Guatemala City 01010, Guatemala

**Keywords:** schwannoma, ulnar, nerve, dual, surgery

## Abstract

We present a compelling case of a 41-year-old woman who presented with paresthesia in the ulnar distribution and a palpable mass near the distal forearm. Examination showed a non-mobile, deep mass with a positive Tinel’s sign. Preoperative magnetic resonance imaging identified a well-defined lesion suggestive of a nerve sheath tumour. Intraoperatively, a second mass in the Guyon canal was discovered. Exploration revealed a second, smaller lesion within the Guyon canal. Both schwannomas were excised, leading to full symptom resolution. This case highlights the importance of a comprehensive diagnostic approach to inform surgical decision-making and ensure optimal patient outcomes.

## Introduction

Schwannomas are the most common tumours of the peripheral nerves, typically benign and well-circumscribed, arising from Schwann cells. They tend to occur in the head, neck and the flexor surfaces of the limbs [1]. Representing ~5% of benign soft tissue tumours, their incidence is ~1 per 100 000 individuals annually. Schwannomas are most frequently diagnosed in individuals aged 30–50, with a slight female predominance. Ulnar nerve involvement, especially distal to the elbow, occurs in 75% of cases and is typically solitary in 90% [1, 2].

These tumours may remain asymptomatic for years. However, growth can lead to nerve compression, causing pain, paresthesia or sensory and motor deficits [3]. Imaging with ultrasound or magnetic resonance imaging (MRI) helps preoperative assessment, but histopathology confirms the diagnosis. Surgery is recommended for symptomatic or cosmetically concerning tumours [3, 4].

## Case report

A 41-year-old woman presented with a one-year history of a growing mass on the medial distal left forearm. She reported paresthesia in the fifth finger, pain upon ulnar deviation of the wrist, and a positive Tinel’s sign, without pain at rest. She denied prior trauma, surgery or comorbidities.

**Figure 1:**
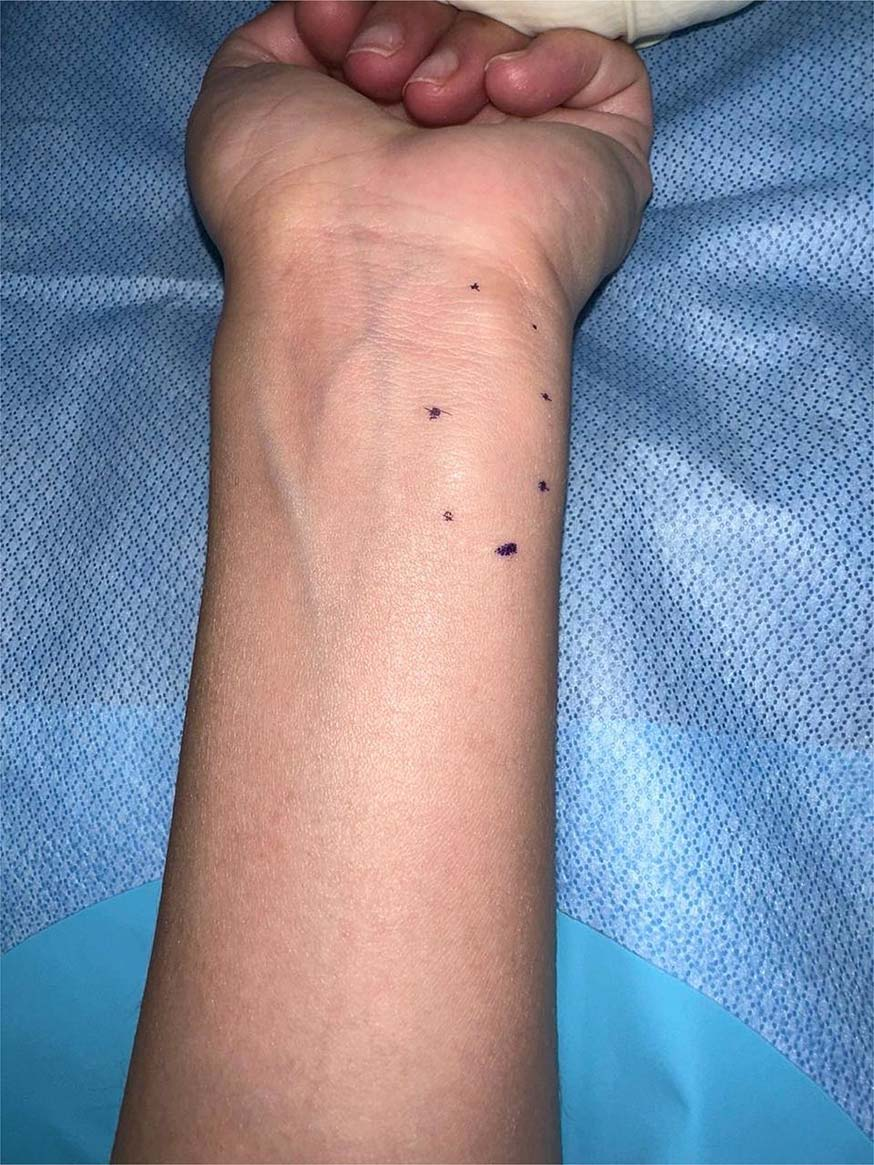
A well-defined, elongated mass measuring ~4 × 3 cm is visible on the ulnar aspect of the distal forearm. The lesion is outlined with small skin markings to delineate its borders.

Physical examination revealed a soft, non-mobile mass on the ulnar side of the distal forearm, near the wrist. It was adherent to deep planes, and there were no skin changes muscle atrophy (Fig. 1).

**Figure 2:**
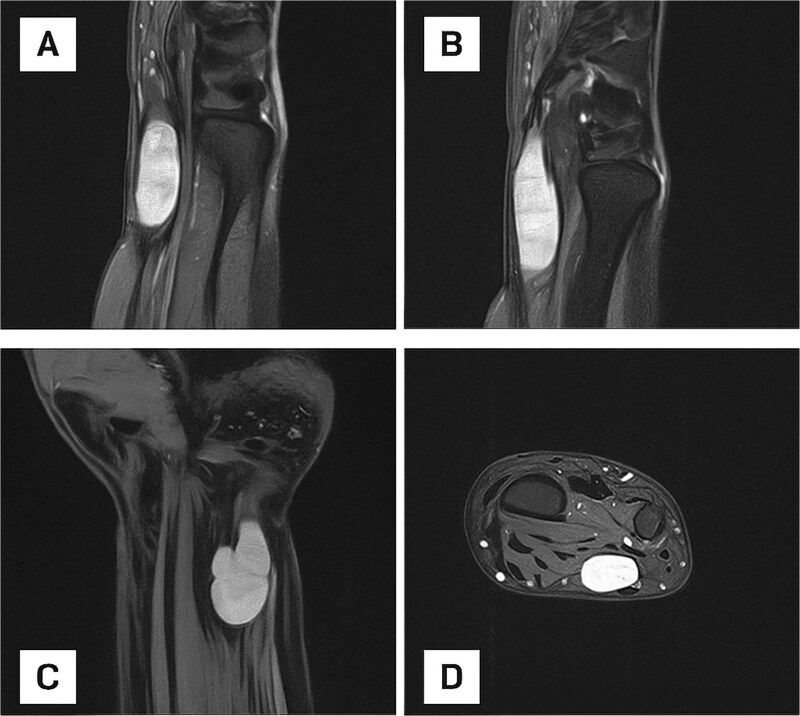
(A) Sagittal MRI scan of the distal forearm demonstrating a well-defined, hyperintense lesion on the ulnar aspect, suggestive of a fluid-rich or soft tissue mass. (B) Sagittal MRI scan depicting an elongated, well-circumscribed hyperintense lesion with no evidence of bone involvement or perilesional edema. (C) Coronal MRI scan showing a lobulated mass, raising suspicion for a nerve sheath tumour or multilobulated ganglion cyst. (D) Axial MRI view confirming an encapsulated lesion within the deep soft tissue of the distal forearm.

MRI was performed to further characterize the lesion. It revealed a well-defined, oval-shaped, hyperintense mass on the ulnar aspect of the distal forearm, adjacent to the ulna (Fig. 2). The lesion appeared encapsulated, suggesting a cystic or soft tissue origin. Coronal sequences demonstrated a lobulated morphology, raising suspicion for a nerve sheath tumour. No bone involvement or edema was seen.

The patient underwent surgical excision under regional anesthesia. A longitudinal incision along the ulnar forearm was made. A well-encapsulated tumour measuring 4 × 3 cm was identified, adherent to but not infiltrating the ulnar nerve. A second lobulated mass measuring 0.5 × 0.5 cm was unexpectedly found inside the Guyon canal. The incision was extended to access and completely excise the second mass.

**Figure 3:**
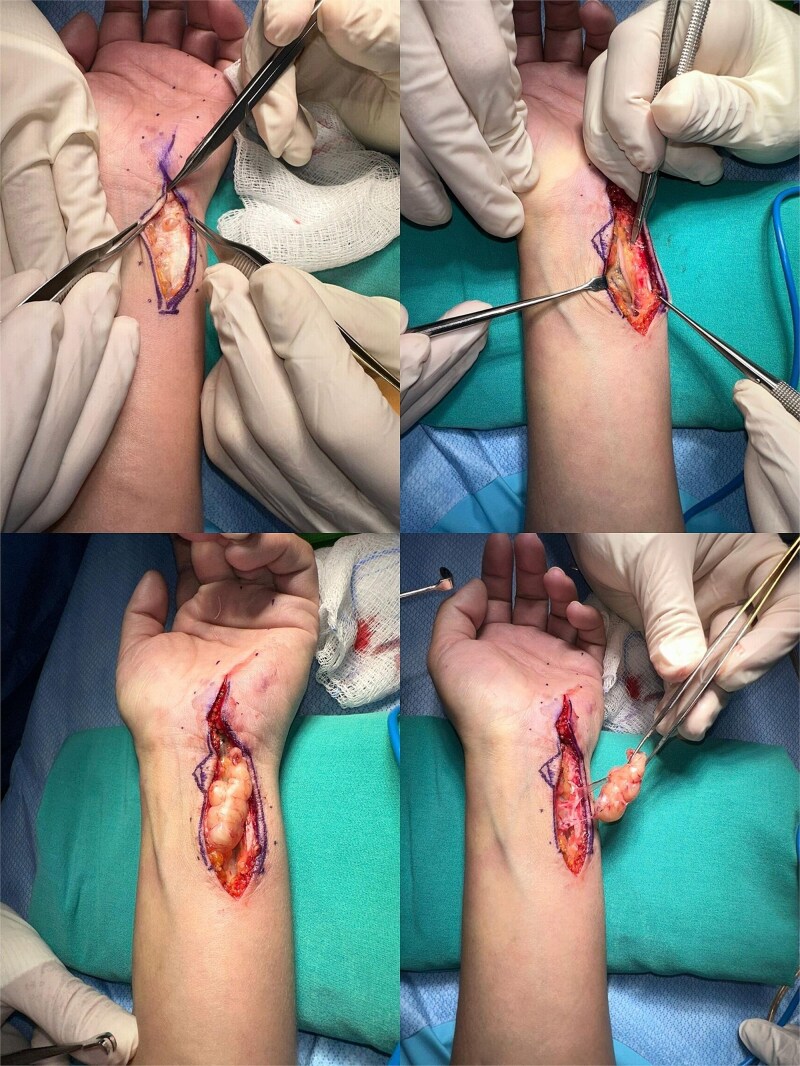
Intraoperative photographs showing surgical exposure and excision of both schwannomas. The primary mass was located along the ulnar aspect of the distal forearm. A second, smaller lesion was identified intraoperatively within the Guyon canal and excised.

**Figure 4:**
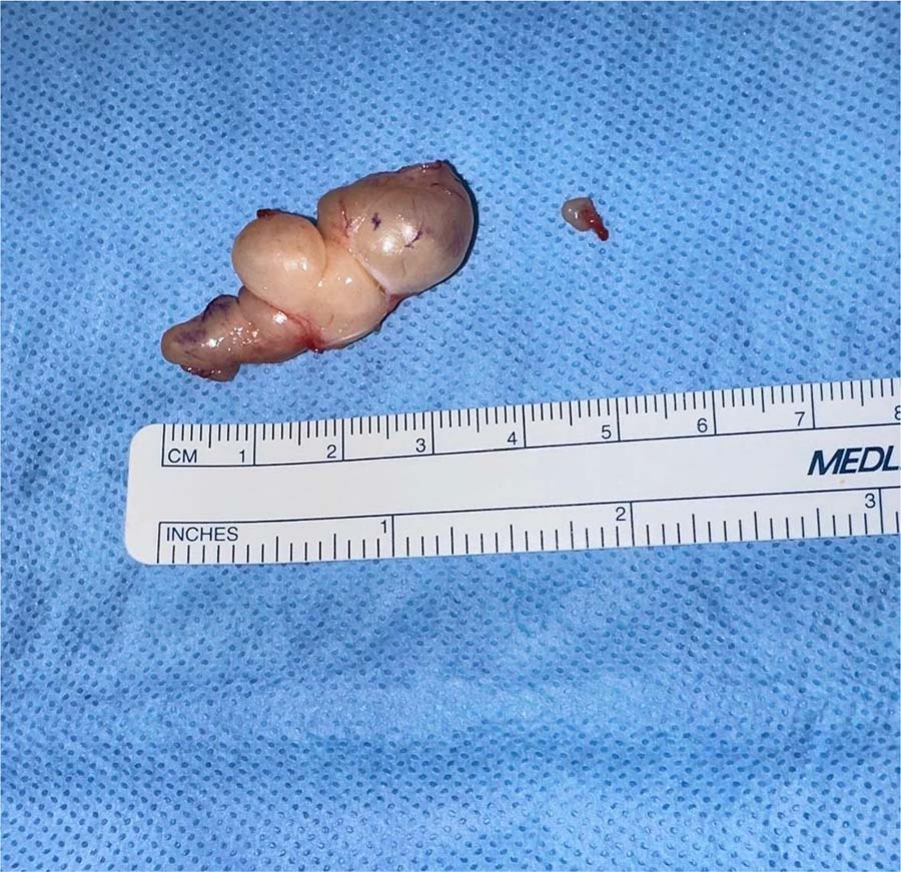
Gross photograph of excised schwannomas. The larger, multilobulated mass (left), measuring ~4 × 3 cm, corresponds to the schwannoma visualized on MRI. The smaller lesion (right), ~0.5 × 0.5 cm, was discovered intraoperatively within the Guyon canal. Both tumours were completely excised.

Both tumours were successfully removed without complications (Figs 3 and 4).

Two to three days after surgery, the paresthesia resolved, and the patient currently has no complaints. The postoperative course was uneventful, with no signs of complications.

## Discussion

Distal ulnar nerve schwannomas are rare, as most peripheral nerve tumours in the forearm involving the median nerve [5]. In this case, symptoms such as fifth finger paresthesia, wrist pain and a palpable mass with positive Tinel’s sign suggested a nerve sheath tumour, with schwannoma being the most likely cause. The clinical challenge in this case lies in the rarity of ulnar nerve schwannomas in the distal forearm and wrist.

MRI imaging revealed a well-defined, hyperintense mass along the ulnar side of the distal forearm, consistent with a schwannoma. This confirmed the presence of a benign nerve sheath tumour, and helped differentiate it from other soft tissue lesions that may mimic peripheral nerve tumours. Interestingly, a second smaller mass located within the Guyon canal was not clearly visualized. This second mass, measuring 0.5 × 0.5 cm, was only discovered intraoperatively. Despite its small size, this lesion was likely a key contributor to the patient’s symptoms, particularly paresthesia in the fifth finger. Despite its modest size, its position in the Guyon canal, where the ulnar nerve passes through a confined anatomical space, made it a significant source of nerve compression.

Histopathological examination of the excised mass confirmed the diagnosis of a benign schwannoma characterized by both Antoni A and Antoni B areas. These findings are typical for schwannomas, with Antoni A regions demonstrating compact, cellular spindle cells arranged in palisades and Antoni B regions showing more hypocellular areas with myxoid stroma. The positive S100 immunohistochemical stain was consistent with schwannoma, further supporting the diagnosis and ruling out other lesions such as neurofibromas or malignant peripheral nerve sheath tumours [6, 7].

Surgical excision was performed, and the primary mass located along the distal forearm was successfully removed. The second mass within the Guyon canal, discovered during surgery, was also excised. This discovery highlights the limitations of preoperative imaging, particularly MRI, which failed to detect the smaller mass. This underscores the importance of intraoperative vigilance and the need for careful exploration of the nerve sheath when dealing with nerve tumours, especially in areas where multiple lesions may be present. The patient experienced complete resolution of her symptoms after surgery, emphasizing the importance of complete tumour removal, including secondary masses that may not be visible on preoperative imaging.

The size and location of schwannomas play a critical role in determining the severity of symptoms [8]. Even a small tumour, like the one found in the Guyon canal, can lead to significant neurogenic symptoms if it compresses vital nerve structures. Smaller tumours in the wrist and forearm may not always present with prominent symptoms until they reach a size that exerts pressure on adjacent structures. In this case, the second mass was small but located in a narrow anatomical space, making it more likely to compress the ulnar nerve and cause symptoms. This highlights the importance of considering even small lesions as potential sources of symptoms in regions with confined anatomical spaces, such as the Guyon canal.

While schwannomas are typically benign, they can cause significant morbidity due to nerve compression. In this case, the prompt and effective management of the schwannomas through surgical excision led to a favorable outcome, with no recurrence or postoperative complications noted during follow-up. However, postoperative monitoring remains crucial, as schwannomas, even when benign, can recur if excision is incomplete or if there is residual tumour tissue left behind in difficult-to-reach anatomical areas. Long-term follow-up is essential to monitor for any recurrence, particularly in complex regions such as the Guyon canal, where small secondary lesions might be missed on imaging. Regular MRI follow-up and clinical evaluations will help ensure that the patient maintains normal function and that any delayed onset of symptoms is detected early.

In conclusion, ulnar nerve schwannomas in the distal forearm and Guyon’s canal are rare benign tumours that present notable diagnostic challenges due to their ability to mimic more common upper extremity conditions. Although clinical presentation often includes neurogenic symptoms and occasionally a palpable mass, the infrequency of these tumours can contribute to delayed or incorrect diagnoses.

High-resolution imaging, particularly MRI, plays a crucial role in accurate localization and preoperative planning, although smaller lesions may not always be detected. Surgical excision remains the treatment of choice, with complete removal of both the primary tumour and any secondary lesions being essential to optimize outcomes.

## References

[1] Hilton DA, Hanemann CO. Schwannomas and their pathogenesis. Brain Pathol 2014;24:205–20. 10.1111/bpa.12125 24450866 PMC8029073

[2] Pertea M, Filip A, Huzum B, et al. Schwannoma of the upper limb: retrospective study of a rare tumor with uncommon locations. Diagnostics (Basel) 2022;12:1319. 10.3390/diagnostics12061319 35741129 PMC9222006

[3] Azaditalab H, Farzan A, Hamdollahzadeh H, et al. Schwannoma of the upper extremity: a clinical series. J Orthop Spine Trauma 2024;10:28–30.

[4] Adani R, Baccarani A, Guidi E, et al. Schwannomas of the upper extremity: diagnosis and treatment. Chir Organi Mov 2008;92:85–8. 10.1007/s12306-008-0049-0 18612585

[5] Morrey LM, Patel S, Lichterman M. A recurrent schwannoma in the left distal ulnar nerve. Cureus 2024;16:e64535. 10.7759/cureus.64535 39144886 PMC11322103

[6] Das S, Dey P, Banerjee AK. Schwannoma and neurofibroma originating from the ulnar nerve in neurofibromatosis: a case report and review of the literature. J Orthop Case Rep 2020;10:47–50. 10.13107/jocr.2020.v10.i05.1780 PMC7487326 32939396

[7] Kabra P, Yadav MVK, Peddamadyam S, et al. Recurrent ulnar nerve schwannoma in the cubital tunnel elbow: a rare presentation and surgical management. *Cureus* 2024;16:e73631. 10.7759/cureus.73631 39677119 PMC11646317

[8] Agarwal A, Chandra A, Jaipal U, et al. Imaging in the diagnosis of ulnar nerve pathologies: a neoteric approach. Insights Imaging 2019;10:37. 10.1186/s13244-019-0714-x 30895491 PMC6426899

